# Five-Year-Old Children’s Working Memory Can Be Improved When Children Act On A Transparent Goal Cue

**DOI:** 10.1038/s41598-019-51869-4

**Published:** 2019-10-25

**Authors:** Christophe Fitamen, Agnès Blaye, Valérie Camos

**Affiliations:** 1 Université de Fribourg, Fribourg, Switzerland; 2 Université d’Aix-Marseille & CNRS-LPC, Marseille, France

**Keywords:** Short-term memory, Working memory, Human behaviour

## Abstract

Working memory is a key component of human cognition and its development throughout childhood a major predictor of cognitive development and school achievement. Noticeably, preschoolers exhibit poor performance in working memory tasks. The present study aimed at testing different means to improve working memory performance in preschoolers. To this aim, we tested the effect of abstract and transparent goal cues in a Brown-Peterson task performed by 4- and 5-year-old preschoolers. If the transparent goal cue helps to better maintain the instructions, it should lead to better memory performance. Moreover, preschoolers had to track, either visually or with their fingers, the goal cue during the retention interval. If the motor activity favors the active engagement of the children in the task, the finger tracking should lead to improvement in memory performance. Our findings were that 5-year-old children benefitted from a transparent goal cue when they acted on it, while 4-year-old children did not show any improvement. These results suggest that working memory performance can be improved in 5-year-old children when the task embeds elements that can scaffold the task goal.

## Introduction

Which parent has never experimented asking her child to pick up a cloth in her bedroom and her child came back without the cloth or never came back? Such a behavior could result from a transitory neglect of the goal while it can be recalled if explicitly required. This phenomenon, named goal neglect, lead to actions inconsistent with the goal even though the goal is not totally forgotten^1^. The critical role of goal maintenance has repeatedly been evidenced in the field of executive control (for reviews, see^2,3^). In contrast, its potential role in working memory has been subject to much less investigation. Yet working memory tasks involve potentially costly goal maintenance during the delay between encoding and recall of the memory items, in particular when a secondary task has to be performed. Inspired by the literature in goal maintenance in task-switching, the aim of the present study was to examine how to improve working memory performance in preschoolers. Indeed, preschoolers are known for their poor working memory performance, and finding ways to help them performing working memory task can inform us on the mechanisms that support their achievement. More specifically, our study aimed at testing two types of manipulations. On the one hand, we examined how the degree of transparency of a goal cue (i.e., contrasting an abstract to a transparent cue) that was introduced between encoding and recall can affect recall performance. On the other hand, we varied the active engagement of the children in the task by introducing a visual or a motor activity between encoding and recall. These manipulations lead to four different conditions performed by 4- and 5-year-old preschoolers to examine how working memory performance was improved.

## Working Memory in Childhood

Working memory plays an important role in children’s development in general and in school achievement in particular. Children with high working memory capacities have better school achievement than children with low capacities (for a review, see^4^). Working memory capacity increases throughout childhood^5–7^, and such an increase is considered as a major source of cognitive development (for a review, see^8^). Different factors have been mentioned as potential sources of working memory development during childhood. Changes in the speed of forgetting and in the processing speed, global increase of cognitive resources and of the size of the focus of attention, acquisition of maintenance strategies and long-term knowledge have been evoked and could jointly underpin working memory development (for a review, see^8^).

Among these different factors, the ability to maintain the goal of the task is rarely considered. If preschoolers’ representation of the goal of a working memory task, which is to maintain information for further recall, is not sufficiently active, this could, at least partially, account for poor performance. Preschoolers are known for having difficulties in maintaining goal in contexts of competing goals, and goal neglect has been frequently mentioned in the executive control literature to account for their poor performance in task switching and inhibition tasks (e.g.^9–13^). Working memory tasks are examples of tasks in which several goals are competing with each other. Because working memory tasks often require to maintain information while performing a concurrent task, participants have to switch between the encoding of the memory items and the concurrent task presented during the retention of these items. At the end of a trial, they also have to switch from the concurrent task to recall. Some models of working memory even suggest that participants have to switch from performing the concurrent task to item maintenance during the retention interval (see^14^). Hence, to achieve a working memory task, there are several competing goals, and memory performance can, at least in part, depend on the efficiency of goals maintenance. This may be particularly difficult for young children.

## The Development of Goal Maintenance

Goal maintenance is crucial in task-switching and inhibition tasks. The importance of goal maintenance in preschoolers was highlighted in the Dimensional Change Card Sorting task (DCCS) in which bidimensional stimuli have to be sorted by one dimension on a pre-switch series of trials and by the other on the post-switch series. The switch of sorting rule necessitates goal updating^15^. Marcovitch, Boseovski, and Knapp^11^ have evidenced preschoolers’ tendency to neglect the new goal in the post-switch phase by contrasting two conditions, one in which a majority of post-switch stimuli were “redundant” cards that were sorted the same for both rules and another condition in which the majority of cards were “conflict” ones, whose sorting depended on the rule at hand. Considering the proportion of sorting errors of conflicts cards on post-switch trials, the findings supported the authors’ hypothesis: The goal neglect is more probable in the “mostly redundant” condition as the sorting of redundant cards provide no incentive to reactivate the post-switch goal, both rules leading to the same correct response. Interestingly, similar effects have been observed in adults when solving conflict tasks, like the Stroop task. These effects correspond to a reduction of the compatibility effect (i.e., difference between performance on compatible -Blue written in blue- vs. incompatible trials -Blue written in red) when lists involve mostly incompatible trials compared to mostly compatible lists. Kane and Engle^16^ interpret these effects as an indication of the goal-supportive context built by the mostly incompatible trials that induce a more frequent reactivation of the goal. In clear contrast, mostly compatible lists favors goal neglect, hence an increase of the compatibility effect.

Another line of evidence for kindergarteners and preschoolers’ difficulties at maintaining tasks goals is to be found in studies testing the benefit of goal-cueing in the resolution of control tasks. Chevalier and Blaye^10^ have shown that 5- and 6-year-olds sorted cards more quickly and more accurately in a DCCS task when the goal cue displayed at the onset of each post-switch trial was transparent (a multicolor ribbon to sort by color) was than arbitrary (a grey square introduced as corresponding to color sorting). The effect of the transparency of the goal cue was replicated in other studies with flexibility tasks^13,17^ and was also observed in inhibitory tasks (e.g., go/no-go like^9^). Task-switch studies in adults and children suggest that participants maintain the current goal in a verbal format^18^. Accordingly, Chevalier and Blaye^10^ revealed that auditory transparent goal cues, suppressing the need for a verbal translation, were even more efficient at supporting goal maintenance than visual ones.

## Goal Maintenance in Working Memory Tasks

Less than a handful of studies have explored the role of goal maintenance in working memory tasks. It is all the more surprising given that studies on executive control suggest that participants who are the most sensitive to the proportion of goal-reminding trials (i.e. conflict trials) are those with the poorest working memory capacity. Hence, it seems plausible that preschoolers’ poor working memory capacity might partially result from difficulties in maintaining goals.

In an already old study, Istomina^19^ compared two settings of assessment of free recall to 3-to-7 year-old children. Children were either involved in a situation similar to a laboratory test condition, or play a shopping game. In the former, lists of items were presented and children had to recall them. In the latter, the lists of items were presented as shopping lists and children had to go to a toy shop and asked for the items to another child playing the merchant. Preschoolers have better recall performance in the play condition than in the laboratory condition. It can be then suggested that the shopping game context have emphasized the task goal (memorizing for further recall) through the provision of a pragmatic relevance to recall. Hence, the overall context of the task can then provide some cueing that would help to maintain the task goal, resulting in improved memory performance.

In a similar game situation, Bertrand and Camos^20^ tested preschoolers’ verbal working memory. The task was also a shopping game in which 4- to 6-year-old children had to memorize lists of verbal items for further recall either immediately after the presentation of the items or after a delay. Moreover, according to conditions, children had to walk straight to the toy shop or to wait seated in the front of the shop during the delay. Results indicated an increase in recall performance at all ages in the walking condition compared to the waiting condition. Among the different interpretations put forward by the authors, one is suggesting that walking being a goal-oriented motor activity, it may have improved goal maintenance in preschoolers and led to better recall performance. Findings of these two studies, in line with studies on the development of executive control, suggest that when children’s memory capacity is assessed in a goal-supportive context, preschoolers’ performance improves.

In contrast, a recent study examining the impact of different goal cues, did not reveal any benefit on preschoolers’ working memory performance evaluated in a lab situation^21^. While the introduction of a visual goal cue in the retention interval did not affect recall performance, an auditory-verbal cue led to poorer performance. This suggests that, unlike studies in task-switching in which verbal transparent goal cues revealed optimal, this format is not adequate in verbal working memory tasks. In fact, verbal cues could interfere with the memory items, because they were verbal, and visually and auditorily presented in Fitamen *et al*.‘s study^21^ as in most verbal working memory tasks in young children. As a consequence, the present study used visual cues to reduce the possible interference with the to-be-maintained material. Further, the two studies that reported improvement in recall performance in preschoolers^19,20^ share the fact that children were actively involved in the task. On the contrary, children in Fitamen *et al*.'s study^21^ were passively seated in front of a computer on which items and cues were presented. Hence, asking children to perform a motor activity oriented toward the goal may be critical in helping them to better maintain the goal, because children would be more actively engaged in the task. The present study tested this hypothesis by contrasting conditions in which children had or not to perform an action on the goal cue in the retention interval.

## The Present Study

In this study, we examined how to improve working memory performance in 4- and 5-year-old preschoolers. For this purpose, we asked children to perform a Brown-Peterson task similar to the tasks used in Fitamen *et al*.‘s study^21^. This working memory task required to maintain information during a retention interval while doing another task, here a tracking task. We deliberately chose a secondary task that captures attention (as in most working memory tasks), but was also embedded in the main task, thereby making the retention interval meaningful. Indeed, as the retention delay corresponded to the time needed by the cue to follow its path towards the location of recall of the memory items (i.e., display of a question mark on the right side of the screen) tracking the cue transformed a void and meaningless delay into a time necessary to accomplish a task-related meaningful activity. One of our aims was to assess whether a visual tracking was enough for children to be engaged in the memory task or whether a stronger engagement through the finger-tracking of the goal cue was preferable. Series of drawings were visually presented one by one on a computer screen with their auditory name. Children had to remember these series for further recall after a retention interval of a few seconds. In this interval, a shape appeared on screen and moved from left to right, the end of its movement corresponding to recall time. The moving shape was either a geometric shape with no explicit link to the memory task or a transparent goal cue (i.e., a school bag to carry the pictures to the recall point). Moreover, children had to track the moving shape either visually or with their finger, the latter requiring a more active motor involvement. The orthogonal crossing of these two variables (cue: abstract vs. transparent, and tracking: visual vs. finger) defined the four experimental conditions. Each child was tested in one of the four conditions, and a control condition similar to the visual tracking of the geometric shape, albeit with a different shape, served as baseline to allow cross-condition comparisons due to the between-subject design of our experimental manipulations.

Extrapolating from findings in executive control tasks, we expected that a transparent cue favors goal maintenance. As a consequence, preschoolers should exhibit better recall performance in the conditions involving a transparent cue. Regarding the way of tracking the moving shape, finger tracking requires more active motor involvement than visual tracking. This should be specifically critical in the transparent goal cue condition. Finger tracking is more demanding than visual tracking, and it should then be more detrimental for memory performance, because attention is more distracting away from the memory items. However, in the case of a transparent cue, the finger tracking could be more beneficial than a visual tracking, because it emphasizes the final goal (i.e. recalling) through the virtual “transport” of the pictures (i.e. memory items) to the recall point. Finger tracking was thus hypothesized to enhance the benefit from a transparent cue in that the motor activity on the cue should provide continuous support to goal maintenance during the full retention interval.

## Results

Performance was measured by the number of words correctly recalled. One 4-year-old and seven 5-year-olds were excluded from the analyses due to ceiling performance. Three 4-year-olds and two 5-year-olds outliers (+/− 2 *SD*) were also excluded from the analyses. Before analyzing recall performance in the experimental conditions, we checked that the groups assigned to the four different conditions did not differ *a priori* by performing an analysis of variance (ANOVA) in each age group on children’s performance in the control condition. Indeed, it was important that the groups created by random assignation did not differ from each other to assess the effect of our experimental manipulations. This revealed that, rather unfortunately, the groups differed in 4-year-olds, *F*(3,75) = 3.48, *p* = 0.02, *η*_*p*_^2^ = 0.12, although not in 5-year-olds, F < 1. To take into account this between-group difference, we introduced the score in the control condition as a covariate, and analyzed the recall performance in experimental conditions through ANCOVAs.

**Figure 1:**
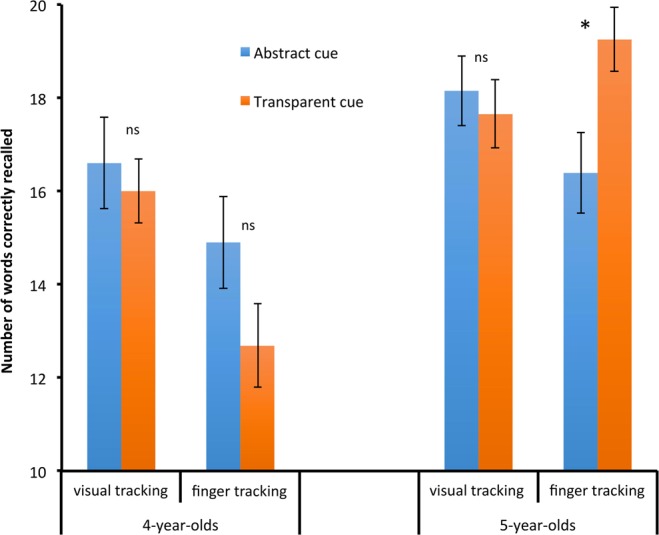
Mean number of words correctly recalled in 4- and 5-year-old children according to cue (abstract vs. transparent) and tracking (visual vs. finger). Y bars represent standard errors.

The ANCOVA on recall performance score in the experimental conditions with Age, Cue and Tracking as between-subjects factors revealed an interaction between the three factors, *F*(1,148) = 6.50, *p* = 0.012, *η*_*p*_^2^ = 0.04. Hence, we performed separate analysis for each age group. In 4-year-olds, the cue effect was never significant, *F* < 1 in the visual-tracking condition and *F*(1,36) = 1.34, *p* = 0.26, *η*_*p*_^2^ = 0.04, in the finger-tracking condition. This led to an absence of Cue x Tracking interaction, *F*(1,74) = 2.24, *p* = 0.14, *η*_*p*_^2^ = 0.03 (Fig. 1). Contrasting with the findings in 4-year-olds, presenting a transparent goal cue led to a significant improvement in 5-year-olds’ recall performance when the children had to track the cue with their fingers, *F*(1,35) = 6.36, *p* = 0.02, *η*_*p*_^2^ = 0.15. This beneficial cue effect vanished in the visual-tracking condition, *F* < 1, resulting in a significant interaction between Cue and Tracking in 5-year-olds, *F*(1,73) = 4.82, *p* = 0.03, *η*_*p*_^2^ = 0.06. (Fig. 1).

## Discussion

The purpose of this study was to improve preschoolers’ working memory performance. To this aim, we analyzed the extent to which supporting goal maintenance with goal cue and actively engaging children in the task could improve preschoolers’ performance in a Brown-Peterson task requiring children to track an animated shape during the retention interval. As in task-switching paradigms, in such working memory tasks, children have to switch between different goals, the encoding goal, the secondary task goal (here tracking), the recall goal, and potentially item maintenance goal during the retention interval (see^14^). Hence, inspired by studies in executive control tasks, we expected that a transparent cue reminding the recall goal could favor its maintenance, leading to better recall performance. Moreover, considering the contrasted results reported in working memory studies that tempted to support goal maintenance^19–21^, we suggested that actively engaging children in the task by asking them to act on the goal cue during the retention interval, like tracking the cue with a finger, could increase the beneficial effect of cue on goal maintenance and recall performance.

Findings in 5-year-olds provided some support to our predictions. While a transparent goal cue did not enhance performance when visually tracked, 5-year-old children significantly improved their recall performance when they had to follow the transparent goal cue with their finger. This result is in line with one hypothesis put forward by Bertrand and Camos^20^ that emphasized the role of a motor activity oriented towards the goal in helping to maintain the goal. It can also be observed that the lack of evidence for a benefit of the transparent cue when children did have to act on it echoes Fitamen *et al*.’s findings^21^. They showed no effect of the introduction (vs. absence) of a cue in working memory tasks in which children remained rather passively in front of a computer screen. Overall, these findings support the idea that preschoolers need to be actively engaged in the task to improve their memory performance. In contrast with their older peers, 4-year-olds did not show any benefit of goal cueing in the visual and finger tracking conditions. This result may seem at odds with the findings in executive control literature, which reports that the youngest children get the most benefit from goal cue^12^. Although further research is needed to support our interpretations, 4-year-olds’ failure to benefit from any of the two goal-cue conditions may be due to an overload introduced by complex instructions. Indeed, the supplementary instructions required to provide its status to the transparent cue, and to present the motor tracking task, were both very demanding for these younger children. Thus, this may have hampered the effect of finger tracking of the goal cue. In other words, introducing the transparent cue involved the addition of an animation before the start of the cue move across the screen. Further, accurately tracking an animated shape with their finger requires fine motor skills that might be specifically demanding on young preschoolers’ attentional resources (e.g.^22^). Hence, the goal-oriented motor task may have oriented 4-year-olds attention away from the recall goal maintenance. By contrast, the working memory improvement observed in 5-year-olds with a goal-oriented tracking echoes findings in the literature on gestural production.

Whereas motor tracking is attentionally demanding for younger children, the benefit of performance from tracking the cue with their finger in 5-year-olds suggest that, as proposed by Golding-Meadows and colleagues^23,24^, motor activity can release attentional resources when it is well in reach of children’s motor abilities. Further, the finger tracking used here is a motor activity of low intensity, and low intensive motor activities have been shown to improve performance contrary to more highly intensive ones (for a review^25^).

An alternative, and more speculative, interpretation of our findings can also be put forward. The contrasted pattern of results between the 4- and 5-year-olds in recall improvement may inform us on differences about the working memory strategies the two age groups are able to implement. Actively tracking a transparent goal cue seems to provide an optimal context to trigger item maintenance strategies (such as verbal rehearsal or attentional refreshing) in 5-year-olds. It could be proposed that these children have these strategies in their repertoire, but, outside a context that scaffold the recall goal, they have difficulties activating them. By contrast, in the 4-year-olds, this context was not enough to trigger item maintenance strategies. This could be due to these strategies still not yet available in the 4-year-olds’ repertoire. The alternative hypothesis of finger-tracking being too demanding for younger children must however be eliminated before any final conclusion.

Although a methodological constraint of the present study does not allow direct age comparisons (see Method section), the contrasted patterns obtained in 4- and 5-year-olds suggest age-related differences in preschool years. More specifically, the present findings suggest that, older preschoolers can improve their working memory performance with adequate manipulations. A goal-oriented motor activity involving a transparent goal cue that remains present during the full retention intervals allows to increase memory performance.

## Conclusion

To conclude, the present study is, to our knowledge, the first to test the conjunctive impact of goal cues and motor activity on preschoolers’ performance in a working memory task. It showed that a transparent goal cue can positively impact working memory performance insofar as it is associated to a motor engagement that requires its continuous tracking. In other words, proposing a goal-oriented gesture on the cue is crucial to boost recall performance. This could have implications for educational settings, which could integrate some motor goal-oriented activity to scaffold memory in young children.

## Method

### Participants

One hundred and seventy preschoolers of 4- (83 children, mean age = 3.7, *SD* = 0.3 year, 37 girls) and 5-years of age (87 children, mean age = 4.7, *SD* = 0.3 year, 41 girls) participated to the experiment. All children were native French speakers, and none was color-blind. Children were schooled in 6 different schools in Marseille (France) and surroundings. Informed consent was received from a parent or legal guardian for each child. Children also gave verbal assent to participate. Although individual demographic information was not collected, most children were Caucasian and from middle to high socioeconomic status backgrounds, reflecting the demographics of the geographical area. Data were collected from January to June 2016. The study was approved by the ethics committee of the University of Aix-Marseille, and was performed in accordance with relevant regulations.

### Material and procedure

The experiment was organized in two 15-minute sessions. To prevent fatigue in the 4-year-olds, a 15-minute pause was introduced between the two sessions. Among the two sessions, one was dedicated to the control condition and the other to one of the four experimental conditions. The order of the conditions (control vs. experimental) was counterbalanced across participants. Three different experimenters (2 women and 1 man) collected data, two of them being blinds to the aim of the experiment. The experiment was built on E-Prime 2.0.10 (Psychology Software Tools) and ran on laptops in a quiet room in the children’s school.

**Figure 2:**
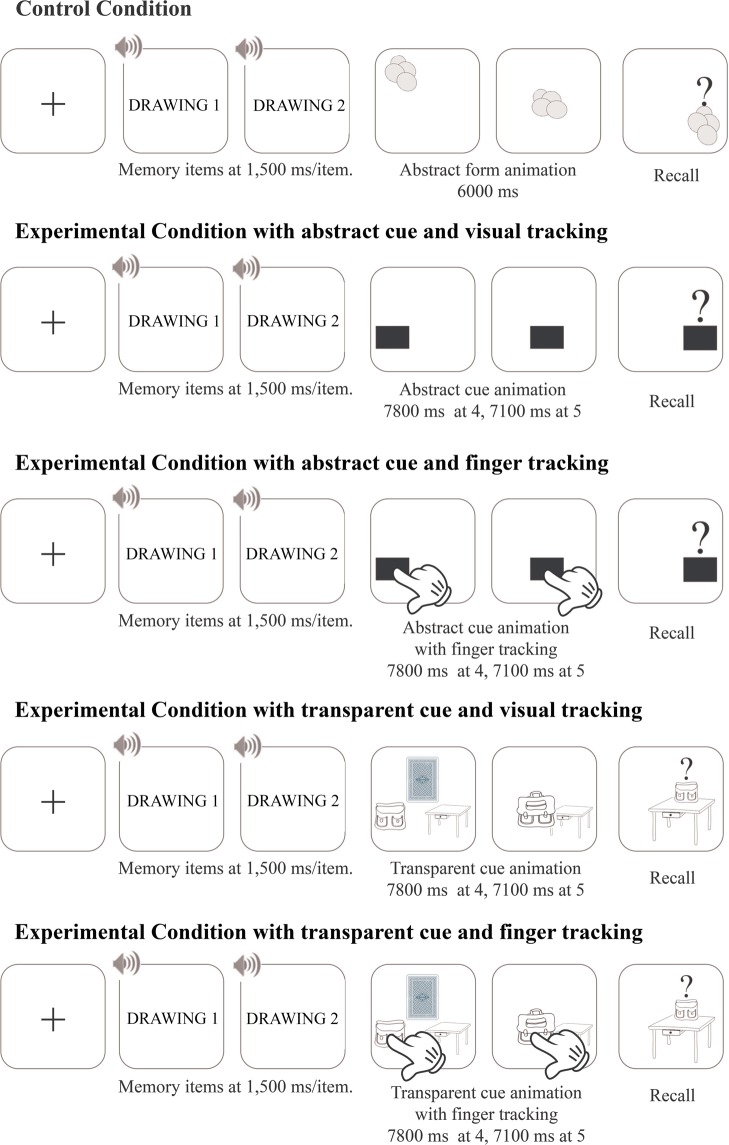
Control condition and the four experimental conditions in which the presentation of memory items was followed by the animation of either an abstract form, an abstract cue or a transparent cue. Depending on conditions, the cue is tracked visually or with one finger. Elements in the animation are not drawn to scale.

In all conditions, children performed a Brown-Peterson task in which they had to memorize the names of animals and objects that were presented visually and auditory (Fig. 2) while tracking a moving shape on the screen during the retention interval. In each condition, twelve series of one to three colored drawings of to-be-remembered items were presented with four series of each length.

Drawings were taken from Rossion and Pourtois^26^, who created colored version of the Snodgrass and Vanderwart^27^ material. The drawings are courtesy of Michael J. Tarr, Center for the Neural Basis of Cognition and Department of Psychology, Carnegie Mellon University, http://www.tarrlab.org/. Each image was selected to exceed 80% of correct denomination at 4 years of age^28^. The name of each memory item was recorded by a male voice and presented simultaneously to its drawing. To reduce proactive interference, two lists of items were created and each of them was assigned to one session. The presentation order of the lists was counterbalanced and the presentation order of the items within a list was randomized.

In all conditions, a black cross, appearing in the center of the screen for 500 ms, signaled the begin of each trial. It was followed by the sequential presentation of the memory items. Drawings were displayed in an invisible 10 cm square at the center of the screen for 1,500 ms. After a 200 ms delay, an animation started and was shown during the entire retention interval (Fig. 2). This animation was different across condition.

In the control condition, a 3 cm × 2.3 cm large gray abstract shape moved at a constant speed following a non-linear path that included 2 turns, starting from the left top corner of the screen. It lasted 6,000 ms delay until the shape reached the right bottom corner of the screen where a question mark appeared to prompt the free recall of memory items (i.e., without any requirement to recall in order of presentation). During the animation, children had to visually follow the abstract form (Fig. 2). The abstract form and the question mark remained on screen until the end of the recall.

Whereas all children did the control condition, they were randomly assigned to one of the four experimental conditions, created by the crossing of 2 types of goal cues (abstract vs. transparent) and two tracking conditions (visually vs. with a finger). The procedure in the experimental conditions was similar to the control condition, except that the animation of the abstract shape was replaced by the animation of a 3 cm × 2.3 cm large black rectangle moving from the left bottom corner of the screen to the opposite corner at a constant speed for the abstract cue condition and by the animation of a 3 cm × 2.3 cm large school bag for the transparent cue condition (Fig. 2). For this latter condition, the drawing of the last memory item in a series was replaced by an illustration of the back of a playing card. The 3.9 cm × 5.5 cm large card was first displayed vertically in the center of the screen. It moved into the bag, circling upward, while swinging 180 degrees to the left. At the same time, its size was gradually reduced to disappear when entering in the bag. This animation lasted 2000ms. Then, the bag was closed and started to move. When it reached the table on the opposite corner, it opened with the apparition of a 1.3 cm × 2.5 cm large question mark just above the bag (Fig. 2). The bag animation lasted 4000 ms. To make clear that the card represented the memory items, the experimenter used real playing cards in the instruction phase, with the same card back and the same initial dimensions than in the animation and some drawings akin to the memory items on the other side.

In all conditions, the experimenter told the child that she was going to see pictures on the screen and that the game was to keep them in her head to recall them later to the experimenter. In the transparent cue conditions, the experimenter further specified during the instructions phase that these pictures were on cards similar to the ones placed in front of her. Moreover, different instructions were given on how to track the cue, as children had track the cue either visually or with their finger. During the instructions phase in the transparent cue condition with visual tracking, the experimenter described the animation while it was running, i.e., the picture went into the bag, then the bag moved to the table, where it finally opened. The experimenter then asked the child to tell him what pictures was in the bag. If the child has some difficulties to answer, the experimenter helped by asking what the child had seen before. In the transparent cue condition with finger tracking, the experimenter also described the animation and told the child that she had to touch the bag and to follow it with her finger. Here, the animation was triggered by the experimenter when the child touched the bag. In the abstract cue condition with visual tracking, the experimenter only told the child to look at the movement of the black rectangle (the term “square” was used to facilitate children’s understanding) and did not further comment on the animation. Once the animation ended, the experimenter asked the child to tell him the pictures she had seen before. In the abstract cue condition with finger tracking, the experimenter indicated to the child to touch and follow the rectangle with a finger. In the same way as in the transparent cue condition with finger tracking, the animation was triggered by the experimenter when the child touched the cue.

We presumed that the time to touch the cue after its presentation on screen would differ across age groups, being longer in the 4-year-olds, and especially in the transparent cue condition, for which there were several elements on screen to be explored. We then tested first children in the transparent cue with finger tracking to evaluate their time to touch the cue. As expected, 4-year-olds took longer (1800 ms on average) than 5-year-olds (1100 ms). Hence, to match the retention interval across the 4 experimental conditions, these average durations were added to the 6000 ms of animation, resulting a total delay of retention of 7,800 ms in 4-year-olds and 7,100 ms in 5-year-olds. Such a design with different retention intervals between age groups would not allow to assess any age-related difference in working memory capacity. However, this is not the aim of this study, as ample evidence has been already gathered on the fact that old children outperformed younger in working memory tasks. The aim of the study relied on the comparison between cue conditions according to the requirement to act on the cue, hence asking for same retention intervals between conditions but within an age group.

Each experimental session was preceded by a training phase. Children had two trials of one memory item and one trial with two items. Also, children involved in the finger tracking conditions received additional training during five trials where they tracked with their finger one object (different in each trial) moving in different directions.

## Data Availability

Data can be asked to the corresponding author.
